# HRM 4.0 and New Managerial Competences Profile: The COMAU Case

**DOI:** 10.3389/fpsyg.2020.578251

**Published:** 2020-11-20

**Authors:** Ezio Fregnan, Silvia Ivaldi, Giuseppe Scaratti

**Affiliations:** ^1^Department of Psychology, Catholic University of the Sacred Heart, Milan, Italy; ^2^Comau S.p.A., Grugliasco, Italy; ^3^Department of Human and Social Sciences, University of Bergamo, Bergamo, Italy

**Keywords:** HRM, E-HRM, digital transformation, social value, corporate responsibility

## Abstract

The digital revolution has generated huge changes in the world of work, with relevant implications for the Human Resources Management (HRM) function. New challenges arise in facing digital work, digital employees, and digital management, such that the connection between new technologies and HRM is now described as electronic HRM (e-HRM). Challenges and connection entail the possibility to review the notion of HRM itself, examining new research perspectives and lines of interpretation following a Critical Management Studies approach, thus developing a more contextualized view in conceiving HRM, a more expansive consideration of stakeholders, and a longer-term perspective in approaching the results of digital transformation and HRM outcomes. The article analyzes a specific organizational case, involving a multinational enterprise, and explores how the case study enhances the understanding of HRM as a social practice embedded in specific situated contexts. Such a conception enables the engagement of multiple rationalities, related to both internal and external stakeholders, overcoming a “mere antiperformance stance” and achieving forms of reconstructive reflexivity concerning the interconnection between the digital age, HRM, and the innovative generation of social value through an authentic corporate responsibility.

## Introduction: Facing The Fourth Industrial Revolution’s Impact on HRM

Undoubtedly, the world is coping with huge changes in culture, society, and the economy that are direct consequences of the digital revolution. Technologies play an essential role in the upcoming Fourth Industrial Revolution, characterized by three main aspects: the increasingly widespread use of Internet, the introduction of artificial intelligence, and the diffusion of automatic learning (Schwab, 2016). Such revolutionary technological changes are transforming the world of work and, consequently, impacting management practices at different levels. The HRM function is strongly involved in coping with the spreading digital age, facing “digital employees,” “digital work,” and “digital employee management” (Strohmeier and Parry, 2014). Thereby, the demand for a strong revision of the traditional approach in conceiving the managerial function, specifically HRM (Janssens and Steyaert, 2009), and in achieving new competences and tools for changing and aligning strategies and activities to these new labor features (Manuti and de Palma, 2017) is warranted.

The research aims to explore the challenge of the 2019 Business Roundtable (a think tank of 200 North American CEOs, founded in 1972) concerning the need to promote better and socially responsible corporate governance. Innovative statements on the purpose of a corporation were agreed by about 200 CEOs of the most important worldwide companies^1^, such as having fair and ethical relations with employees and suppliers, supporting the communities embracing sustainable practices across businesses, and generating social and long-term value for shareholders as a meaningful manifestation of an authentic corporate responsibility. Such a new stance, compared to the neo-liberal approach, entails implications for a different way in conceiving management, specifically in the field of HRM, which represents a peculiar context in which social purposes unfold among tensions, contradictions, and situated practices. At stake is a different way in conceiving and studying the field of HRM as a set of social practices, embedded in a global/local economic, political, and sociocultural context. The authors refer to Janssens and Steyaert (2009), whose study reviews the notion of HRM by examining new research perspectives and lines of interpretation following a Critical Management Studies approach (Alvesson and Willmott, 1992, 1996; Alvesson and Deetz, 2000; Alvesson and Kärreman, 2007; Alvesson et al., 2008). Compared to a more traditional approach to HRM (scientific and rational; legitimate and authoritative; right to control others in the interests of efficiency and productivity), the authors suggest a different vision (complex and constructed; ideological and privileging; political, exploitative and open to critical thinking and moral debate), better suited to match the challenging dimensions that underpin the connection between digital technologies and HRM.

Janssens and Steyaert (2009) emphasize a practice-oriented research, claiming for alternative interpretations and new perspectives, suggesting to study HRM as a set of social practices. Such a suggestion entails going beyond a traditional inquiry of individual variables in studying HRM and its relationship with organizational process, achieving the political nature that underlines the HRM discourse. The authors argue that a more pluralistic perspective, analyzing real practices of HRM, “further provides ways to allow professionals to develop more skills approaches” (Janssens and Steyaert, 2009, p. 146). Their suggestion of “bringing the employee back” into HRM studies implies both micro- and macrolevels of analysis and to consider “the inherent pluralism of work life” (Janssens and Steyaert, 2009, p. 146), focusing more explicitly on the implications of new forms of work for employees without assuming a harmony of interests, also taking into consideration the broader political–economic forces influencing the way in which work is managed. They also claim for the introduction of new vocabulary, going beyond the dominance of the economic logic and developing different, alternative forms of reconstructive reflexivity around the HRM debate.

The most fruitful direction for the field of HRM is studying HRM practices as social practices: researches should try to understand how in a specific organization or context HRM is produced through connecting and interweaving various discourses, micropractices, and rhetorical strategies. Indeed, HRM practice encompasses techniques, actions, and strategies inside the micro- and macrocontext of societal and political–economic circumstances and can be acknowledged as an outcome of human interpretations, conflicts, and confusions, including agency and subjectivity. In this sense, HRM study also entails a reflexive stance, connecting “different perspectives and voices…approaching the field of HRM as a heterotopic text where various academic languages, concepts and practices are assembled through more complex, social processes of arguing, influencing and experimenting” (Janssens and Steyaert, 2009, p. 152). In this article, the authors address these suggestions using a case study as a proper and coherent approach in order to examine some HR practices developed in a situated context, specifically the enhancement of new managerial competences in a 4.0 Industrial Revolution scenario. Similarly, Bondarouk and Brewster (2016) underline the challenge for a different and more contextualized point of view in conceiving HRM, a more expansive consideration of stakeholders, and a longer-term perspective in approaching the results of digital transformation. Arguing that “*good* HRM consists of policies and actions that work for the survival and success of firms in the long run, rather than just creating short-term returns to shareholders” (Bondarouk and Brewster, 2016, p. 2657), they criticize “the widespread silence of the academic community on HRM’s culpability for contributing to the global economic crisis that began in 2008 and the failure of HRM specialists to suggest ways forward” (Bondarouk and Brewster, 2016, p. 2,568), aiming at a sustainable HRM that better reflects practitioners’ reality.

The authors highlight the impact that the strong transformations of work in the twenty-first century have been generating on HRM, claiming for a less neo-liberal and more comprehensive view of this topic and seeking for new e-HRM research and practice. They plea for approaches able to give voice to those involved in the HRM organizational life, since “different situations have different cultures and different institutions” (Bondarouk and Brewster, 2016, p. 2656). The need to listen to line managers and employees depends on the fact that “the use of e-HRM is not necessarily binding for all stakeholders, and different target groups develop their own ways of coping with e-HRM, then organizations often face the situation when individual’s technological enthusiasm and decision to first use e-HRM is different from the decision to enact and continuously work with e-HRM” (Bondarouk and Brewster, 2016 p. 2662). The authors suggest as a matter for empirical investigation the issue of how far companies are able to introduce e-HRM perspective both in practices and employee perceptions as well as in policies. Summing up the above theoretical background and frameworks, they underline the need for a new way in conceiving and studying HRM: more contextualized, open to different perspectives and voices, oriented toward effective practices, facing the heterotopic text of HRM field, seeking for its capability to generate social and long-term values, and reshaping a new way of corporate responsibility.

Following such a perspective, in this article, the authors aim to understand the implications for those involved in HRM organizational life facing the new technologies yielded by 4.0 Industrial Revolution. Therefore, the authors focus on a specific situation related to the case study of a high-tech company in order to enhance the inquiry of the currently transformed HRM context. The authors selected a specific context, an multinational enterprise (MNE), aiming to detect the different configuration of digital sensitivity among internal stakeholders working inside a complex organizational workplace, following the theoretical request to listen to managers and employees, “bringing employees back,” as a specific issue of the practices the article analyzes. The way people use and apply technologies is related to the context, while *per se* technologies tend to be boundaryless. In the chosen specific context, the connection between local and universal features of technologies is related to the diffused organizational culture of agile work and human management. Regarding the long-term outcomes, the authors addressed a specific project of the firm that considers not only the subjects within the organizational boundaries as stakeholders but also the larger society (in this case teachers, families, students, and schools).

The authors seek to answer the following research questions:

• What different technological dispositions (digital sensitivity) of internal stakeholders can be detected and acknowledged in order to develop goals regarding e-HRM, their e-HRM tasks, and their involvement in e-HRM processes;
• How an innovative organizational culture related to the diffusion and use of digital innovation can be promoted and spread;
• Why a specific practice, related to an innovative project, can be seen as an authentic and not sugar-coated way for a sustainable corporate responsibility, which generates value for the society.

In order to explore and provide empirical data related to the research questions, the article examines a case study. The three questions are sustained by the convergent attention to the context, the multiple stakeholders, and the long-term outcomes highlighted by Bondarouk and Brewster (2016) and refer to the solicitations of Janssens and Steyaert (2009) to approach HRM as a social practice, giving relevance to different levels and voices. The article is organized as follows. The first section begins by examining the connection between the digital age, the effects on institutional, social, and labor market fields, and the implications for innovative forms of HRM, which underpin the knowledge questions addressed. The Comau case study is then presented, focusing on three initiatives developed to point out the situated relationship between new technological developments and a specific HRM context, connecting the methodological choices with the research questions. The conclusions are drawn in the final section, where the findings are illustrated; some considerations, comments, limitations, and hints for future research are provided.

## HRM Challenges at Stake

The digital age is recognized as being a relevant phenomenon at a global level, having generated significant changes in different life environments, from culture to society and the economy, giving rise to important questions about the pros and cons and the benefits and disadvantages of the rapid spread of technological and digital innovation (Bennett et al., 2008; Nawaz and Kundi, 2010). Regarding the effects on society and institutions, Schwab (2016) highlights how the high connectivity provided by new technologies allows solutions able to develop both potentials (closeness and connection between people and institutions; simplification of hierarchies and bureaucracy in the production and diffusion of knowledge; more acknowledged and autonomous people and workers; more skilled and talented people) and risks (increase in the gap due to the unequal distribution of resources; growth of precariousness). In the labor market, two opposite effects on employment are spreading: the destructive effect, which leads to replacement of the labor force by pushing workers toward unemployment, and the capitalization effect, which by increasing the demand for new goods and services, leads to the creation of new jobs but also new companies and markets (Makridakis, 2017). The challenges generated by the 4.0 scenario asked for workers with new skills concerning problem solving and communication and also the HRM profession are expected to enable new ways of participation and of doing business, creating new products and services and providing new ways of organizing the workforce—all at a distance (Bondarouk and Brewster, 2016, p. 2662). The effect on competences (Ruël and Bondarouk, 2014; Bondarouk et al., 2015; Hecklau et al., 2016) highlights the need to develop both transversal skills (from execution to entrepreneurship) and digital capabilities for the interaction between humans and machines. The impact of the digital revolution specifically involves companies aiming to keep business competitive by becoming part of this new paradigm (Strohmeier and Parry, 2014). The players of the Fourth Industrial Revolution are organizations deeply involved in a transformation process that is both a powerful opportunity and a tough challenge. Although digital technologies offer possibilities to reduce costs, speed operational processes, and enhance collaboration among HR stakeholders (Strohmeier, 2009; Parry, 2011), they also yield disadvantages like digital divide, hyperconnectivity, reduction in face-to-face contact, and the loss of relevance facing technical professionals (Parry and Tyson, 2011). Regarding the current organizational scenario transformed by 4.0 Industrial Revolution and the connected new technologies, Bondarouk and Brewster (2016) remind to conceive e-HRM as the territory of “all integration mechanisms and all HRM content shared via IT that aim to make HRM processes distinctive and consistent, more efficient, high in quality and which create long-term opportunities within and across organizations for targeted users” (Bondarouk and Brewster, 2016, p. 2659), hoping that the HRM research could contribute to improve the understanding of this phenomenon.

In the literature, the connection between new technologies and HRM is described as electronic HRM (e-HRM), which is what Ruël et al. (2004, p. 368) have defined as “a way of implementing HRM strategies, policies, and practices in organizations through the conscious and direct support of and/or with the full use of channels based on web-technologies.” Coping with this emerging scenario, the HRM function itself is disoriented, seeking a new configuration, both theoretical and practical. At stake is the possibility to enhance a different model of HRM, going beyond the dominant neo-liberal view in which the purpose of the firm is solely and primarily to maximize the shareholders’ value (Stout, 2012; Bondarouk and Brewster, 2016). Janssens and Steyaert (2009) advocate that a re(constructive) reflexivity (Alvesson et al., 2008) is needed for theorizing HRM. They propose to go beyond an idealized notion of HRM, considering the material and situated conditions in which HRM is practiced. In this direction, it becomes possible to reshape a realistic link between HRM and organizational processes, which is not taken for granted since it is characterized by conflicting goals and interests, tackling the political features of the employment relationship. A more pluralistic perspective is requested in order to overcome a too narrow vision based on strict economic criteria rather than social values. Such a perspective entails conceiving HRM as a social practice embedded in specific situated contexts, spreading the concept of outcome toward the notion of sustainable generation of value in a more complex understanding.

A common feature underlined in several contributions is that all organizations tackling digital transformations must lead their people into a new business culture, characterized by a massive use of digital technologies and by a set of technical as well as mental skills, to systematically acquire, process, produce, and use digital information (e.g., Nawaz and Kundi, 2010). Hence, the challenge for HRM is to rethink the way to interact with new expectations, attitudes, and values while coping with a changing workforce and reshaping work content and professional competences. In order to cope with increasing transformations, HRM has the chance to develop new ways for enhancing organizational contexts based on sustainability and value generation (Strohmeier, 2007, 2009), thus facing the dilemma of creating new possibilities or proposing a form of neo-Taylorism exasperated by technological devices. The challenge of a sustainable HRM and corporate social responsibility (Strohmeier, 2007, 2009; Bondarouk and Brewster, 2016) does influence the competences (Hecklau et al., 2016; Plumanns et al., 2017), knowledge (Simons et al., 2017), and learning (Erol et al., 2016) that are required by managers. The outlined theoretical framework points out HRM as a project of social accomplishment in which the microlevel of the organizational processes and the internal relationships are intertwined and where the macrocontext is related to the societal and political–economic historical environment. Hence, the need for a new research in the field of HRM, as Janssens and Steyaert (2009) claimed, arguing for approaching the field of HRM as a heterotopic text encompassing languages, concepts, and practices.

## Research Design, Context, and Methodological Approach

Undertaking the study of the complex and articulated e-HRM world, the authors addressed both the three main key elements, highlighted by Bondarouk and Brewster (2016), and the multilevel plural voices stance related to HRM practice, solicitated by Janssens and Steyaert (2009), selecting an emblematic case and applying a practice-based approach. In relation to Bondarouk and Brewster’s hints (context-driven research, implication of stakeholders, and long-term perspective in studying HRM), the architecture of the research allowed to consider:

• The context, taking into account the relevant differences and variations of external and internal conditions and circumstances (people, technology, values, laws, market, and business conditions), assuming the specific environment of an MNE: “Different sizes of firms and firms in different sectors of the economy have different HRM” (Bondarouk and Brewster, 2016, p. 2655);
• The stakeholders, internal and external employees, and social partners, overcoming the implicit paradigm in HRM that “its sole purpose is ultimately to improve financial returns to the owners of the business” (Bondarouk and Brewster, 2016, p. 2657);
• The long-term outcomes in order to enhance benefits and value for all the society seeking “the survival and success of firms in the long run, rather than just creating short-term returns to shareholders” (Bondarouk and Brewster, 2016, Bondarouk and Brewster, 2657).

The authors follow the practice-based research approach outlined by Janssens and Steyaert (2009) to develop a case study for a deeper understanding of meaningful experiences in facing the HRM digital innovation. The Comau case was selected for three relevant reasons. First, because Comau, as an innovative high-tech and robotic company and as an MNE, is facing the challenge of a new way in conceiving and practicing HRM, tackling with external and internal pressure due to the different situations, multiple cultures, and plural institutional subjects. Therefore, it can represent an emblematic, albeit not exclusive, example for an in-depth understanding of the object of study. Comau is the Italian leader in the robotic industry whose headquarters are located in Grugliasco, close to Turin, and fully integrated in the Mirafiori area, one of the most important European centers for the automotive industry. In this location, the FCA Group still has its main production plants (specifically Maserati, Fiat, and Jeep production). Thus, Comau is part of the FCA Group. Even if Comau is an independent corporation (S.p.A.), the control of the company falls on FCA. Moreover, this legal aspect is reflected in policies, procedures, and organization issues where the footprint of the FCA Group is perceived.

Comau currently has more than 9,000 employees worldwide with 15 manufacturing plants and 34 locations. Moreover, five innovation centers are spread across Europe, Asia, North America, and South America. In 1984, the company started its expansion program abroad, establishing different subsidiaries in China, Russia, and South America. The process continued in the 1990s, with the acquisition of companies around the world (France, Germany, Argentina, Brazil, United States, Mexico). In 1997, Fiat acquired all the subsidiaries of the company, formally becoming the holder of the group. As far as the organizational structure is concerned, Comau has four main business units: automation systems, powertrain, robotics, and services. Moreover, aerospace and green consulting (E-Comau) have been created in the last 10 years. Comau is also divided by regional area, with four main areas: EMEA, NAFTA, APAC, and LATAM. Therefore, the organizational matrix is quite complex and is currently under management review to better respond to market requirements. The company is not just selling large and fully integrated production lines for the automotive industry (even if it remains the largest market); it also sells robots, powertrain cells, and services. Robots are focused on the manufacturing industry (especially pick and place robots, welding robots, and agile robots). However, a new market is education, and Comau has just launched a new robot called e.DO for educational institutions (high schools and universities).

Second, the articulated investment in enhancing a different and innovative managerial culture and competence profile conveys interesting empirical evidence in understanding how a new idea of HRM is translated into practice. The investment in e-HRM and the use of digital technologies, coping with complex, plural, and articulated organizational configurations, has become crucial as well as strategic for the corporate HRM, hence the challenge of enhancing a transversal organizational culture able to match both digital competences and soft skills in an integrated view. Comau defined a set of competences 4.0, which incorporate data analytics and technology proficiency with others that cannot be automated:

• Critical thinking as the in-depth analysis of facts to form a judgment. Critical thinking as self-directed, self-disciplined, self-monitored, and self-corrective thinking;
• Cognitive flexibility as the ability to adjust one’s thinking, shifting from old situations to new ones. It is the ability to overcome usual responses or thoughts and thus adapting them to new situations;
• Open-mindedness as receptiveness to new ideas. It relates to the way in which people approach the views and knowledge of others and the willingness to take new viewpoints seriously;
• Complex problem solving as identifying complex problems and reviewing the related information to develop and evaluate options for the implementation of solutions. It is the ability to solve new, ill-defined problems in a complex, real-world setting;
• Creativity as a process of becoming sensitive to problems, deficiencies, gaps in knowledge, missing elements, disharmonies, and so on; identifying the difficulty; searching for solutions, making guesses or formulating hypotheses about the deficiencies; testing and retesting these hypotheses, possibly modifying and retesting them again; finally communicating the results;
• Cooperative networking as sharing knowledge and expertise. Autonomy and independency are combined with the advantage of learning from others and being able to modify the way one works;
• Social intelligence as the ability to relate to others in an efficient, constructive, and socially compatible way;
• Emotional intelligence as the capability of individuals to recognize their own emotions and those belonging to others, to use the emotional knowledge to guide thinking and behavior, and to manage and/or adjust emotions adapting to environments or to achieve one’s goals;
• Judgment as the ability to make reasonable decisions or come to sensible conclusions;
• Decision making as the process of selecting a logical choice from the available options, weighting the positive and negative aspects of each option and considering all the alternatives. A decision maker is able to forecast the outcome of each option as well and, based on all these items, to determine which option is the best for that particular situation;
• Influence as the ability to produce a significant effect on someone or something. If someone influences someone else, they are changing a person or thing in an indirect but relevant way.

The above values and dimensions underpin the declared *humanufacturing* (a crasis between humanity and manufacturing) organizational culture of Comau. In order to transfer such meaningful statements into a rooted, collective, and transversal mindset and attitude, the Comau HRM developed different initiatives and paths, which are the expression of an innovative way to conceive the HRM function and its outcomes. The company designed a Change Model process to better lead its own strategic processes. Comau’s Change Model shows some affinities with the Design Thinking approach and consists of four main steps: (1) active listening, (2) diagnosis, (3) modeling, and (4) tool creation. The listening phase is fully dedicated to listening to the voice of employees in order to understand their points of view and define the best strategy to start a change.

Third, the authors had the possibility to achieve a research agreement with this company due to the belonging and role played by one of them in that firm^2^. The authors of the article shaped and monitored the following three research paths, which allowed an in-depth analysis of the managerial practice in use and constituted the specific embedded contexts for gathering empirical data relevant for the research questions inquired:

a) A survey involving Comau employees worldwide who work in functions impacted by digital transformation;
b) A digital assessment dedicated to the first lines of the company plus 70 key people worldwide who can contribute to stimulating and spreading the digital revolution within the company;
c) A pilot project to promote an innovative training approach for enhancing learning among young students using the e.DO Robot. The project, called Robo-School, was designed by four HR managers of the organization, in collaboration with the schools involved and the representatives of two Italian universities.

From a methodological perspective, the authors took into consideration the above three research paths in order to detect the following: the different configuration of digital sensitivity among the internal stakeholders as a key feature for developing tailored HRM practices (for example, promoting different training for multiple targets); the role middle managers and “ambassadors” could play in spreading and promoting an innovative organizational culture related to the diffusion and use of digital innovation; and empirical data of the customer satisfaction’s result about an innovative educational project as empirical evidence of the capability to generate social values and corporate responsibility. Exploring the digital culture in Comau, selected as specific MNE context, allowed to interpret the Janssens and Steyaert (2009) suggestion to provide plural levels of inquiry and listening to multiple voices. The authors in the following paragraphs briefly highlight the connection between the three research paths and the related research questions.

### The Digital Knowledge Survey for Digital Sensitivity

The first research question refers to possible different technological dispositions (digital sensitivity) of internal stakeholders in relation to their involvement in e-HRM processes. The tool used for gathering data was a survey (see Appendix 1) chosen as a consistent opportunity to achieve proper and coherent empirical knowledge about the above question since its main purposes were to (1) create awareness about digital transformation, (2) collect quantitative data, and (3) define the “state of the art” of Digital culture in Comau. The survey involved 3,585 Comau employees (worldwide managers and middle managers), considered as key persons close to the line and able to detect diffused knowledge embedded in daily practices and activities. In this early phase, the population excluded Blue Collars, HR/Legal/Finance/Internal Audit/Purchasing/Sales and Marketing/Security Safety Facilities.

The Digital Knowledge Survey aimed to explore cluster dimensions related to:

• *Digital skills and knowledge self-evaluation* [MES/PLM/MRP/ERP Systems, Programming Languages, Cloud, Communication API, Sensors Knowledge, Edge Computing, Internet of Things Infrastructure, Data Management (analysis, aggregation, security, database), Machine Learning Algorithms, Artificial Intelligence Techniques, Digital Twin Concepts, ICT Infrastructure (Networking, Wireless, Communication Protocols); GUI Design];
• *Digital areas of interest* (Apps Development/Programming Languages, Internet of Things, Cloud Technology, Data Management, Machine Learning/Artificial Intelligence, Digital User Experience, Digital Trends Outlook, Mobility and Connectivity); and
• Personal feelings about Digital (passionate, curious, low interest).

### The Digital Assessment for Ambassadors’ Involvement in Promoting Innovation

The second research question concerns the ways for promoting and spreading an innovative organizational culture related to the diffusion and use of digital innovation. A key role in Comau HRM processes was assigned to middle managers as “ambassadors” and boundaries spanners of such a diffusion. In order to identify the more suitable people, Comau—taking advantage from its partnership with Mercer, global leader in HR solutions—prepared a tailor-made Digital Assessment. Mercer, using a copyrighted approach and its international benchmark of managerial profiles (the database of Mercer client companies, for reasons of confidentiality, remains private), assessed 80 Comau employees—the first lines of the company plus 70 key people worldwide—in order to provide a Digital Profile Map and to measure their Digital Quotient Index. Both map and index entail the exploration of five important skills (information, innovation, communication and collaboration, cyber safety, and content creation), identified as relevant cultural and psychological dispositions for sustaining the spread of the digital innovation path. The profiles with higher levels for these capabilities were selected for the promotion of the innovative organizational culture, giving importance to the combination of communicative/collaborative stance with the knowledge of the innovative digital contents. The assumption was that communicative competences together with mastery of contents could better support conversations and negotiations needed for the promotion of digital innovation.

### The Robo-School Project With e.DO for Authentic Social Responsibility

The third research question concerns why a specific innovative project can be seen as a way for enhancing an authentic corporate responsibility, able to generate long-term values for society and promote diffused digital culture. The Robo-School project is a meaningful example of how advanced technologies can be used to develop a new way of learning, capable of integrating and supporting traditional teaching tools and methods together with and alongside teachers. In particular, the application of e.DO was intended to: (1) stimulate interest among students; (2) facilitate learning through effective application and practice; (3) connect theoretical contents with everyday real life; (4) encourage active participation, collaboration, and inclusion; and (5) develop both technical competences (connected to the contents of the subjects proposed) and transversal competences. Therefore, the objective of Robo-School is not to ask students to learn robotics but to interact with a robot as a tool to learn traditional subjects (like mathematics and art) in a more interactive, practice-based and collaborative way. In fact, as technology becomes more and more relevant, there will be a growing demand for “human skills” alongside technical and digital ones. Robo-School basically consisted in the implementation of two different modules: the first one focused on mathematics (named “geometry with robots”) and the second one on art (named “the inventiveness of Leonardo”). The two modules were divided in different steps: a sensorial experience (first interaction between students and the robot), the introduction of subjects (presentation of the contents), a fun-applicative experience (practical activities with the robot), and lesson learned (final test based on contents and analysis of the learning experience). The modules were delivered within the schools while a Comau expert facilitated the activities with e.DO. The project is connected to corporate responsibility since it involves internal and external stakeholders in order to generate collective social value, related to the possibility of enhancing the quality and the effectiveness of learning at different levels. The authors adopted a qualitative approach, using a survey and in-depth interviews, to highlight the effectiveness of the project from an external point of view (being the internal one testified by the enthusiasm and the passion of managers and practitioners involved, together with the relevant investment, also an economical one, of the firm): the customer satisfaction data offer a relevant empirical evidence of the convergent acknowledgment and perception of the social and collective value generated by the project.

## Key Findings and Discussion

In this section, the authors describe the main results for each of the three research paths. While for the first two research paths were used traditional methods of descriptive statistics for the data analysis, for the discursive data coming from survey and interviews conducted with teachers and students, the authors used both a phenomenological and semiotic perspective (Mininni et al., 2014; Mininni and Manuti, 2017) and a ricoeurian hermeneutic orientation (Bartunek and Louis, 1996; Cunliffe and Locke, 2019), seeking for the meanings and representations they deliver.

### Findings of the Survey

**FIGURE 1:**
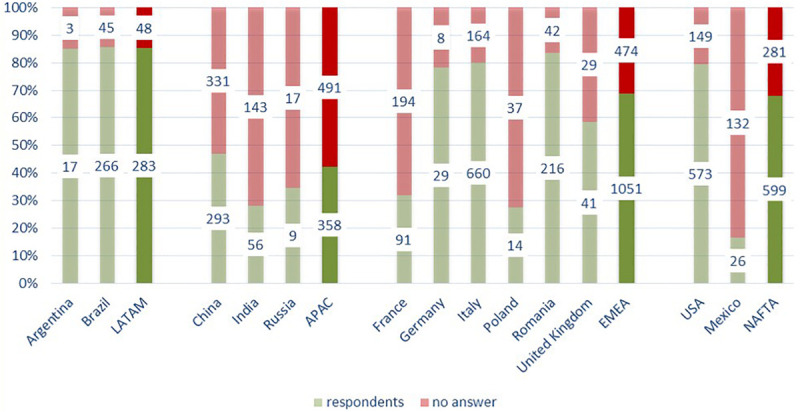
Participation rate.

**FIGURE 2:**
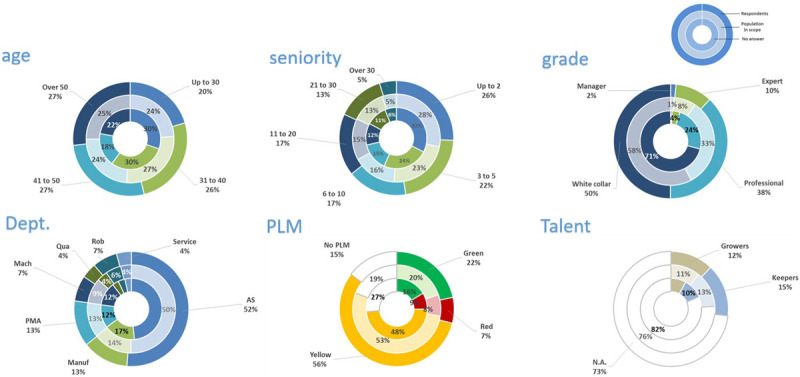
Participation description.

The overall response rate was 64%. A total of 2,291 participants (out of 3,585) around the world answered the questions, which provided data for several relevant findings. From the graph (Figure 1, *Participation rate*), it can be noted that the APAC region scored the lowest rate of response, while LATAM scored the best rate. The EMEA and NAFTA regions showed different levels of involvement. Regarding the participant description (Figure 2, *Participation description*), the survey took into account six dimensions: (1) Age, (2) Seniority, (3) Grade, (4) Department, (5) PLM, meaning the results of the Performance and Leadership Management system adopted worldwide to assess FCA employees, and (6) Talent. Concerning the age, younger than 30 participated relatively less while 40–50 years old participants participated the most, relatively speaking.

**FIGURE 3:**
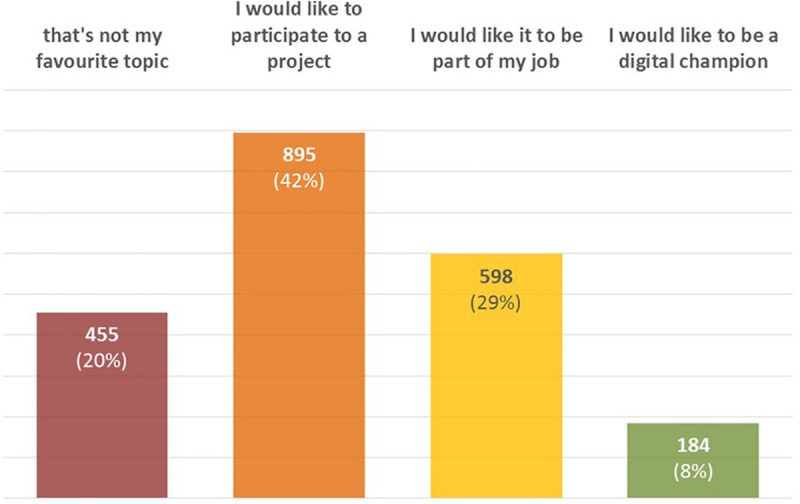
Participants’ availability to participate.

These tests revealed some interesting findings, mainly related to three themes:

I First of all, 80% of respondents would like to participate in digital activities (Figure 3, *Participants’ availability to participate*): digital transformation is a thrilling phenomenon and people want to be part of the change.

**FIGURE 4:**
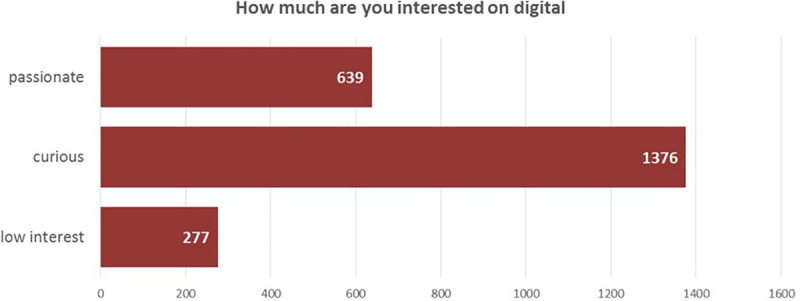
Participants’ interest in the Digital Transformation.

II Second, “taking a picture” of the passions and knowledge that Comau employees have is an effective way to identify the unspoken digital skills within the company.
  a. Starting from the area of interest, participants expressed their curiosity regarding the main phenomenon of the digital revolution: (Apps Development/Programming Languages; Internet of things; Cloud Technology; Data Management; Machine Learning/Artificial Intelligence; Digital User Experience; Digital Trends Outlook; Mobility and Connectivity) and their personal feeling about digital (passionate, curious, low interest).
  b. The results (Figure 4, *Participants’ interest in the Digital Transformation*) show a great number of curious (1,376 participants) and 639 passionate people. The latter are mostly located in Italy, aged between 30 and 50 years. The passionate ones are mostly White Collars; they work in the Automation System Department and their rank in the PLM evaluation system is yellow.

  c. IoT, Artificial Intelligence, and mobile connectivity are considered as the most interesting digital enablers.
III The knowledge level of digital subjects was measured by means of a self-assessment system that mapped the following topics: MES/PLM/MRP/ERP Systems, Programming Languages, Cloud, Communication API, Sensors Knowledge, Edge Computing, Internet of Things Infrastructure, Data Management (analysis, aggregation, security, database), Machine Learning Algorithms, Artificial Intelligence Techniques, Digital Twin Concepts, ICT Infrastructure (Networking, Wireless, Communication Protocols), GUI Design.

**FIGURE 5:**
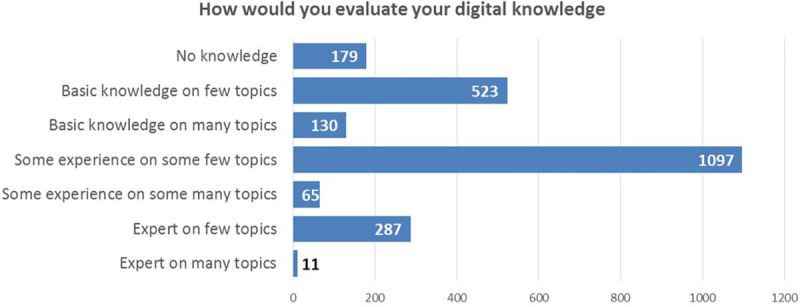
Participants’ knowledge of Digital topics.

The results (Figure 5, *Participants knowledge of Digital topics*) showed that a large part of the participants has some experience in a few of the topics (1,097/2,291). The greatest expertise is about MES/PLM/MRP/ERP Systems, Programming Languages, and Sensors Knowledge. These “subject matter experts” are mostly located in Italy, aged between 31 and 40. They are mostly Professionals and White Collars; they work in the Automation System Department.

**FIGURE 6:**
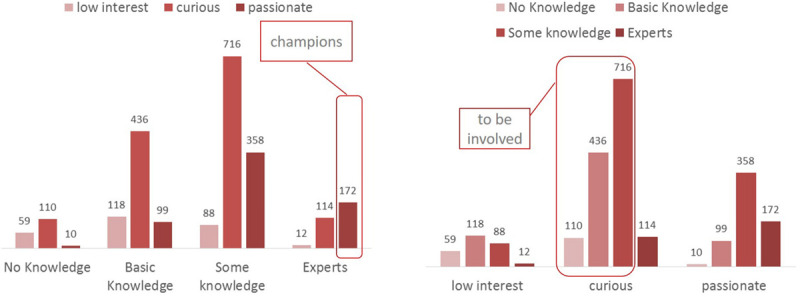
Potential digital champions and curious within the Comau population.

**FIGURE 7:**
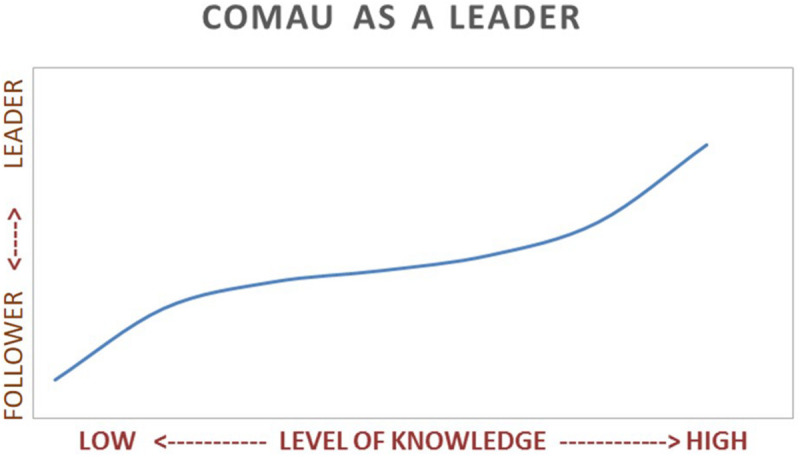
The perception as leader in the Digital Transformation.

The most remarkable consideration that emerges from the data is that the mix of passion and knowledge owned by the people is a real and valuable asset to start a change as complex as the digital revolution itself. Significantly, the survey identified 172 potential digital champions and 1,262 people ready to be involved. Comau will leverage them to start the digital change program because they have the motivation, the skills, and the disposition to diffuse the use of new technologies (Figure 6, *Potential digital champions and curious within the Comau population*). This insight goes along with the fact that Comau’s perception as leader in the digital transformation playground increases with the technical proficiency of the participants. The graph (Figure 7, *The perception as leader in the digital transformation*) shows that the higher the level of knowledge, the greater the ambition to be a digital leader. The data in Figure 7 provide an interesting insight concerning the first research question, in particular the champions and curious for their high level of digital sensitivity, representing a relevant leverage for promoting the employee involvement in e-HRM processes, even though the sample is not representative of all organizational contexts.

### Findings of Digital Assessment

The Digital Profile Map detects four digital profiles that are distributed in the digital change curve:

∘ *Digital Strangers*, who are not interested in the digital revolution and who refuse to use digital technologies;
∘ *Digital Natives*, who were born after the widespread adoption of digital technology and grew up in close contact with those technologies;
∘ *Digital Philosophers*, who were born before the digital revolution but fully understand and support its potential. They do not use technologies, and they will need to develop skills and capabilities to reach the Digital Ambassador level;
∘ *Digital Ambassadors* feel that digital is their natural ecosystem. Their role is to develop skills and capabilities to reduce the digital divide within their organizations.

**FIGURE 8:**
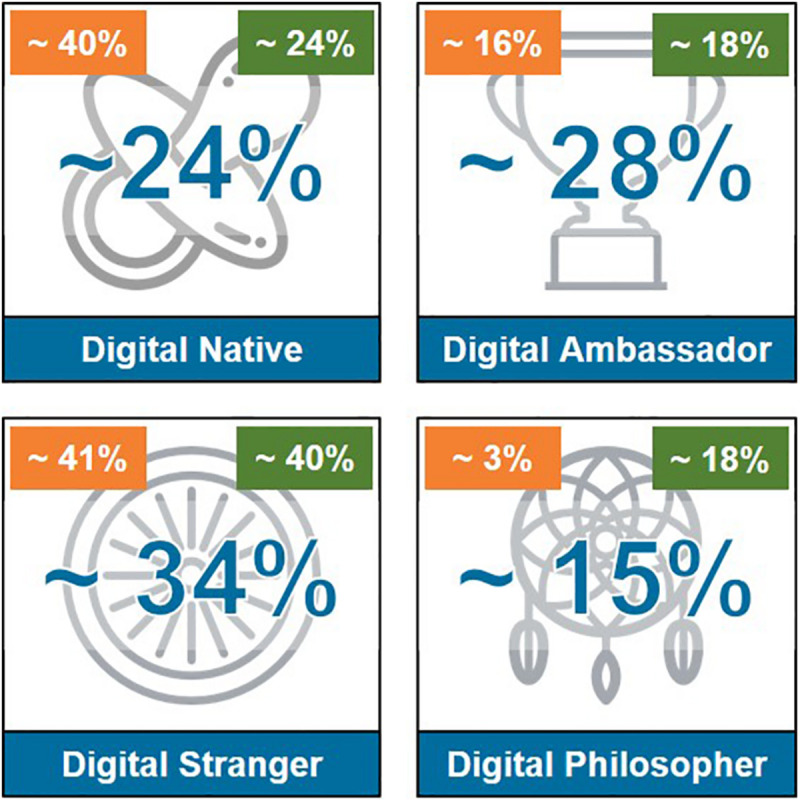
Digital Profile Map.

**FIGURE 9:**
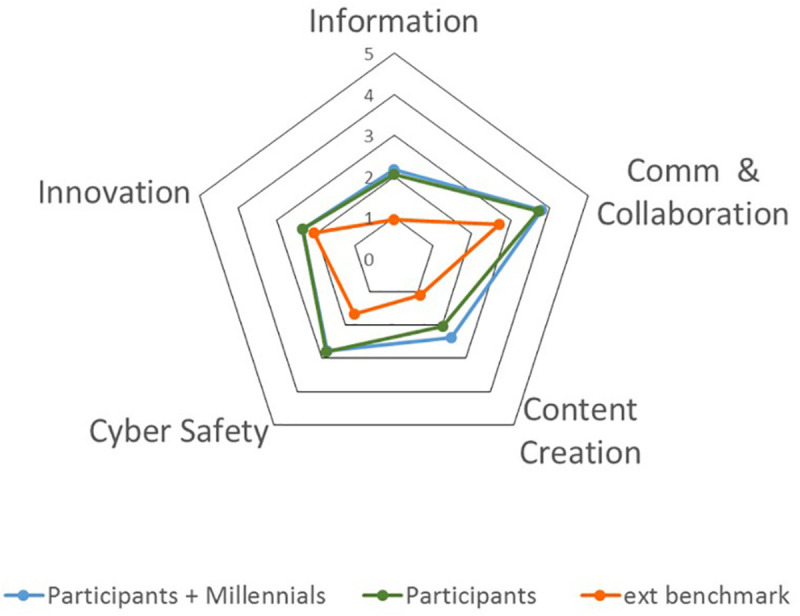
Digital Skills average results.

**FIGURE 10:**
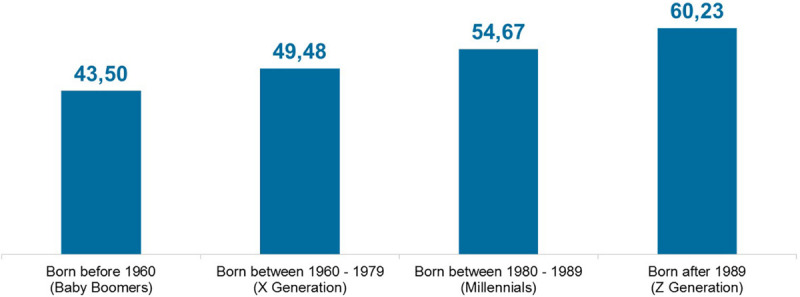
Digital Quotient Index.

The assessment showed (Figure 8, *Digital Profile Map; in the middle Comau values, at the right top corner Millennial values, at the left top corner benchmark values*) that the highest percentage of the assessed participants are Digital Strangers, but they are balanced by Digital Ambassadors and Digital Natives. In the figure, on the top left corner are highlighted the benchmark values related to the Mercer database (not public for copyright issues), while at the top right corner, the Millennials’ values related to the same benchmarks; in the middle are presented the Comau values. There is an interesting presence of Digital Ambassadors who are not Millennials, meaning that first lines and key people born before 1980 are aware of the potential of the digital transformation for business. An interesting observation to emerge from the data comparison concerns two relevant differences: the company is “older” than the benchmarks, and Digital Philosophers are much more present within the organization than outside. The assessment was focused on five main dimensions: Information, Innovation, Communication and Collaboration, Cyber Safety, and Content Creation. These dimensions represent the most relevant skills that the digital age needs. The average results (Figure 9, *Digital Skills average results*) showed a higher mastery of Communication and Collaboration and Innovation skills than the benchmark. In general, average results are higher than benchmark results, and this is a strength for the organization. Finally, the assessment results enabled the measurement of the “Digital Quotient Index” (DQI), which compares the average values of the assessment unbundled in different age targets: baby boomers, born before 1960; Generation X, born between 1960 and 1979; Millennials, born between 1980 and 1989; Generation Z, born after 1989. As can be observed from the graph (Figure 10, *Digital Quotient Index*), the trend shows how DQI is strongly influenced by age, with a digital orientation that is clearly higher for Millennials and Generation Z.

Such evidence represents an important starting point for the next phase of the Comau digital change program. In fact, the company will create a global network of digital ambassadors and enthusiasts that will become the platform for designing, creating, managing, and measuring initiatives to spread the digital culture within the company. The first step will be a quick assessment of the 172 Digital Champions to integrate them within Digital Teams in order to start the change with the support and the engagement of the employees in the first phase. The program will also be promoted by a proper communication plan about current and future Comau Digital activities to spread more awareness within the company. The data collected through the assessment highlight not only the age as an influent feature that can produce different digital capabilities (see the DQI trend) but also the relevance of digital ambassadors and natives. The acquired relevance endorses what Bondarouk and Brewster (2016) underlined about how different target groups can develop their own ways of coping with e-HRM. Concerning the second question, the involvement of digital ambassadors from line managers and employees confirms how the enactment and continuously work with e-HRM is strongly related to dedicated figures, whose role is to sustain, spread, and promote innovative organizational culture related to the diffusion and use of digital innovation.

### Findings of the Robo-School Project

The pilot project involved 38 schools in Piemonte, Italy (14 primary schools and 11 middle schools) for a total of 112 classes, 2,372 students and 120 teachers. It was implemented in the period between March and April 2017. At the end of each module, students had to complete a final test for assessing the experience. A survey and qualitative interviews to the students and the teachers involved in the project were delivered in order to understand if the experience had favored the acquisition and emergence of both technical and transversal knowledge and competences. Four items were assessed: whole appreciation of the experience, learning, competence usefulness, and engagement. The findings refer to the project’s effectiveness and support the practiced and not only declared intention to generate value for multiple stakeholders (in this case, schools, teachers, and students). Offering customer satisfaction details provides empirical evidence related to the capability of the project to generate social value (Comau SpA, 2018; e. Do, 2018).

The questionnaires do not show differences between the different types of schools. It was worth proposing the same questions to teachers and students of different educational stages so that the authors had the possibility to start from the same basis and proceed with a final comparison. For each question, there was a Likert scale of four lengths ranging from 1 (insufficient) to 4 (excellent). The scale, consisting of four possible options, forces those who fill out the questionnaire to make a negative (grades 1 and 2) or positive (grades 3 and 4) assessment. The last one was an open question. In the following tables, the authors present the high level of satisfaction achieved by the project both from teachers and students: that is a not-taken-for-granted result in the logic of value generation in the long-term perspective. Table 1 (*Overall Results – Teachers*) shows the average values *–* for each educational stage *–* of the questions proposed to the teachers. Table 2 (*Overall Results – Students*) shows the average values related to the students. In relation to the qualitative interviews (sample composed by 15 teachers and 60 students), the authors used a semiotic and hermeneutic lens to analyze the qualitative and discursive transcribed material, seeking for some emerging relevant meanings suitable to highlight long-term values generated by the investment in digital innovation. Collaboration, engagement, and funny experience were transversal dimensions analyzed, while the metaphor of the “bridge” emerged as a recurrent picture conveying aspects of connection and integration [bridging between past and future; human and robot/technical artifacts; individual and social; teachers and learners; engagement and fun (the label fun derives from Latin language *divertere*, which means both having pleasure and achieving different, divergent views); cognitive and affective dimensions). Some emblematic excerpts, taken from the interviews with the teachers (Gender/Subject/School Degree), are presented below as they convey aspects and features that characterize the meaning they give to the project (in *italic* the authors’ highlights:

**TABLE 1:** Overall results—teachers.

	**Satisfaction**	**Learning**	**Competence usefulness**	**Engagement**
Primary school	4.00	3.98	4.00	4.00
Middle school (first grade)	3.97	3.76	4.00	4.00
Middle school (second grade)	3.97	3.73	4.00	4.00
Average evaluation by teachers	3.98	3.82	4.00	4.00

**TABLE 2:** Overall results—students.

	**Satisfaction**	**Learning**	**Competence usefulness**	**Engagement**
Primary school	3.93	3.78	3.92	3.90
Middle school (first grade)	3.87	3.65	3.88	3.83
Middle school (second grade)	3.82	3.53	3.88	3.86
Average evaluation by students	3.87	3.65	3.89	3.86

➣ Primary School

“*Innovative strategy* to provide and learn the basics of geometry.” (Male, S.T.E.M., Primary School)

“*Happy combination* of technological and mathematical skills through the game experience: if I enjoy myself and do [things], I learn!” (Female, S.T.E.M., Primary School)

“Illuminating, an example of *modern teaching*. I could observe citizenship skills in children who are hardly observable in the classroom.” (Female, S.T.E.M., Primary School)

➣ Middle School (First Grade)

“Addictive, interesting from an *educational* point of view because it involves a lot of transversal knowledge.” (Female, S.T.E.M., Middle School, First Grade)

“*Captivating and engaging*; precious opportunity for the students.” (Male, S.T.E.M., Middle School, First Grade)

“Very *useful*, also *to reinforce* what has been learned during the curricular activity.” (Male, S.T.E.M., Middle School, First Grade)

“I also really appreciated *the aspect of group work*, which brought into play the ability to collaborate and interact with peers. Thanks, and congratulations!” (Female, S.T.E.M., Middle School, First Grade)

“360° immersive activity. Notable ideas. Problem solving and *cooperation skills*.” (Male, S.T.E.M., Middle School, First Grade)

➣ Middle School (Second Grade)

“Very *captivating approach* and excellent group-class management.” (Female, S.T.E.M., Middle School, Second Grade)

“*Synthesis between* algebra, geometry and technology. Very useful to introduce the Cartesian plan and the straight line.” (Male, S.T.E.M., Middle School, Second Grade)

“Addictive to the nth degree!!! I found it very useful to work with “school” subjects with the support of *objects so loved by students as robots*.” (Female, S.T.E.M., Middle School, Second Grade)

“*Active, collaborative, creative learning*. Very stimulating, didactically effective activity. The hours have flown, capturing the students’ attention in an innovative way.” (Female, S.T.E.M., Middle School, Second Grade)

“Finally, applied mathematics.” (Female, S.T.E.M., Middle School, Second Grade)

“*Stimulation to openness*. Another way to treat known topics.” (Male, S.T.E.M., Middle School, Second Grade)

“An activity that has been perfectly *linked to the activity carried out in the classroom*. Great!” (Female, S.T.E.M., Middle School, Second Grade).

As can be observed, teachers highlight the value generated by the project in relation to different aspects: the educational one, enabling processes of collaborative learning that support and integrate the more traditional individual cognitive process; the innovation of the teaching setting, which encompasses contents, tools, and relations among people; and the interaction with new incoming technologies as robots. In addition, the students (Gender/School Degree) expressed their remarks about the e.DO experience, as the following excerpts allow to underline [in *italic* the authors’ highlights]:

➣ Primary School

“I liked this activity and I would like *to do it again with my friends*. Thank you!” (Male, Primary School)

“I enjoyed this experience so much! For the secondary school I don’t know where to go, but if it exists, I’ll go to *a robotics school*. Thank you.” (Male, Primary School)

“Great fun and also responsibility because we used three unique pieces in the world.” “*I had fun*, it intrigued me and I *discovered things* I didn’t know.” (Female, Primary School)

“It was *fantastic, fun* and at the same time I discovered new things with math and geometry, I also learned how to use a robot.” (Female, Primary School)

➣ Middle School (First Grade)

“Interesting and *very fun* to see the robot making movements.” (Female, Middle School, First Grade)

“An ancient-modern experience between *robots and Leonardo da Vinci*.” (Male, Middle School, First Grade)

“For me, it was *a lot of fun* and educational too, an experience I would like to repeat. I enjoyed interacting with the robots.” (Female, Middle School, First Grade)

“Reviewing topics in a constructive and fun way. Fantastic!” (Male, Middle School, First Grade)

➣ Middle School (Second grade)

“Association of mathematics in robotics in an *engaging way*.” (Female, Middle School, Second Grade)

“A very interactive and *engaging lesson*, very interesting from the point of view of both robotics and art.” (Male, Middle School, Second Grade)

“*Funny, engaging, enjoyable* and competitive. I had fun and I found this experience very useful.” (Female, Middle School, Second Grade)

“A very interesting experience *to approach the world of robotics* and most likely that of a near future.” (Male, Middle School, Second Grade)

“It was like taking a *step into the future*, amazing” (Male, Middle School, Second Grade).

The students stress the value of the possibility to learn as a funny, enjoyable, and engaging experience, bridging fun with engagement, the past with present and future (Leonardo da Vinci and step into the future), human and technologies (approaching the world of robotics). As a final result, the students—through the participation in the project—had the opportunity not only to improve their knowledge related to the issues involved (mathematics and art) but also to experience themselves in the application of specific soft skills, like those of collaboration, negotiation, creativity, and problem solving.

Concerning the third question, the e.DO robot experience is in the authors’ perspective a relevant example of reinterpreting an authentic generation of value, not only for internal but also for external stakeholders, reshaping a new way of corporate social responsibility. Involving different people and stakeholders (Comau engineers and practitioners, teachers, students, schools, educational managers) in negotiated activities, the project matches technology with humans, providing a remarkable learning opportunity and testing the potential of educational impact for society at large. The findings show the effectiveness and the satisfaction for the people involved, since the project yields a new emerging culture focused on innovation and new technologies (robots) that can enhance both processes of contents learning (S.T.E.M. education) and core skills (as collaboration and networking) that students have to perform in order to achieve results. Such a result is a not-taken-for-granted collective value, related to the educational challenge for future generations facing a new technological environment. A meaningful clue about the generated value is that the project was extended (in 2017–2018 Fondazione Agnelli hosted more than 2,400 students in its spaces) with the enrichment of new contents.

As a whole, the findings highlight how the process of e-HRM is enacted in a contextual scenario. Referring to the context of e-HRM, Bondarouk and Brewster (2016) depicted three possible situations: the inertia-based e-HRM enactment, in which HRM professionals, line managers, and employees cope with e-HRM trying to maintain the “old-fashioned” way, preserving the existing technological circumstances; the mutual adjustment enactment, characterized by groups of users that strive to refine their “old” face-to-face HRM practices introducing IT possibilities; and the improvisation-based enactment of e-HRM, with a strong adoption and use of e-HRM applications connected to a great involvement of managers and users in the enactment process and the adjustments it requires. The authors claim that the more advanced, interconnected, and smart e-HRM becomes, the more diverse the involvement of all stakeholders can be expected. The Comau case highlights a possible intertwinement of such different scenarios, with the prevalence of processes of mutual adjustment and refinement of existing performance, as well as the improvisation of new methods, and a less and circumscribed presence of inertia.

In relation to the research questions, about the first one, the findings highlight how the connection between new technologies and HRM has to face differences in technological cognitive frames among the different internal stakeholders. The digital sensitivity varies from one digital generation to another, requiring the corporate Comau HRM to face the situation in which the individuals’ technological enthusiasm and decision to first use e-HRM is different from the decision to enact and continuously work with e-HRM. For what concerns the second question, the role of Ambassadors becomes relevant and crucial, coping with fragmented situations, as Comau’s decision to invest in the Digital Champions to integrate them in Digital Teams clearly points out. Comau’s digital change program, whose purpose is to start the change with both the support and the engagement of the employees, is an emblematic clue of the attention that has to be paid to internal stakeholders in a specific organizational context, like a MNE. A future indicator of the successful implementation of skillful and task-consistent operations will be the application of the new adopted digital practices by the targeted employees. As Bondarouk and Brewster (2016) remark, the implementation is completed only when users are contentedly working with IT and have acquired the necessary skills to master and fully understand it, achieving the objective of a stable use of an e-HRM technology, rather than the stabilization of the technology itself.

The prominent and decisive role attributed to the users in the adoption of digital practices relies on the belief that various stakeholders engaging in implementation exert social influence in order to change the pattern of technology use. In this perspective, Comau is an interesting context, since the e-HRM deployment itself is a result of HRM managerial decision making. Investment in organizational culture and technological engagement emblematically highlights how a firm has to cope with its owners, employees, customers, and communities as stakeholders in HRM. Regarding the third question, the e.DO Robo-School project constitutes a meaningful example of the external stakeholders’ consideration and the intention to generate value in a long-term perspective. The success of the project is given by the combination of multiple drivers: the wide range of players who have worked in synergy to design and deliver the modules (Comau managers and employees, school teachers and students cooperated to shape and enrich the experience); the design of the modules as a balanced mix among content, action, and relation-driven learning, considering as a priority educational (not financial) issues; the integration of disciplines (each module is focused on a specific subject but adopts technology, robotics, and soft skills to be effective); and the gamification lens (asking the students how leveraging on fun and game elements in order to be more involved and participative).

Hence, the engagement of multiple rationalities related to both internal and external stakeholders and achieving forms of reconstructive reflexivity, concerning the interconnection between the digital age, HRM, and the innovative generation of social value through an authentic corporate responsibility. Indeed, investing in such an object and target requires rethinking who the HR managers are asked to be: not just rational agents who organize roles, tasks, and activities but also (and above all) responsive and responsible authors, meaning that they, along with others, actually construct and shape social and organizational realities (Cunliffe, 2014), like the promotion of an innovative digital culture in the Comau case, hither the chance to go beyond the dominant neo-liberal view that maximizes the shareholders’ value seeking for sustainable stakeholder social value (Stout, 2012; Bondarouk and Brewster, 2016). The three research paths and the connected findings outline how HRM is embedded in a specific situated context and which practices are socially developed. Therefore, the possibility to understand and interpret HRM as a lever for generating social value not only inside the specific organization but also outside of it, creating an impact on society at different levels, as in the case of educational aims of the e.DO experience (Fenzi et al., 2013; Pinto et al., 2018).

## Conclusion

Inspired by Janssens and Steyaert (2009) and their approach to HRM as a “heterotopic text” and by Bondarouk and Brewster (2016) with their hint for new ways in conceiving HRM, the authors focused on some emblematic initiatives of HRM developed in Comau, a specific organizational context, that conveyed different voices and languages. This choice is coherent with the theoretical background adopted by the authors, related to the e-HRM field that underlines the need for more contextualized ways in studying the HRM issue, adopting a practice-oriented approach and pluralistic levels of analysis in order to explore the complexity of the dimensions at stake, as the transformation of managerial competences and culture facing the digital innovation conveyed by 4.0 Industrial Revolution. The research questions addressed have been undertaken through the analysis of the Comau case, gathering empirical data that are connected to the conceptual framework, since the analyzed situated research paths are focused on specific contextualized practices that a specific and emblematic organization develops, coping with the digital innovation and its impact on HRM organizational life. Regarding the first question, related to the acknowledgment of different digital sensitivity among the internal multiple organizational players, the case study underlines the necessity to enhance articulated and target-tailored forms of involvement for all stakeholders, detecting their technological proficiency and disposition. The need to face the existing differences entails an investment, which cannot be taken for granted, in knowledge gathering and people management processes. Including employees and managers in studying the interface between HRM and new technology represents a different approach in coping with innovative HRM perspectives. Perhaps, the awareness of differences in digital sensitivity can become one of the new KPIs in order to measure the impact of HRM in generating value, going beyond the too narrow focus on the financial indicators and the implicit paradigm of the financial return as the sole purpose of the owners (Paauwe, 2004).

Regarding the second question, among the ways for promoting and spreading an innovative organizational culture related to the diffusion and use of digital innovation, the role of key persons to be selected and involved as “ambassadors” appears crucial and decisive in order to promote a social and negotiated influence, changing the pattern of technology use. At stake is neither an illusorily manipulation nor a top–down pressure to change but a long-term process of organizational learning and sustainable transformation of usual practices/routines, looking for new and more suitable ones. The last question, related to the possibility to consider a specific innovative project as a proper example of sustainable corporate responsibility, finds a meaningful answer in the Comau e.DO robot project, a promising and emblematic manifestation of the difference existing between a stakeholder approach rather than a shareholder one, due to the involvement of internal and external stakeholders in the practiced effort (and not only declared intention) to generate social an collective value, in this case related to the educational challenge for new generations facing the digital innovation era. It also represents a practical example of the possibility to rethink the conception of the HRM function, assuming alternative perspectives compared with the only economic focus in measuring the HRM outcomes. Sustainable HRM and looking toward a sustainable HRM (Ehnert and Harry, 2011; Taylor et al., 2012) appear as promising and successful new labels, to be studied in depth in their implications in further researches on the future of HRM models. The authors’ observations have several implications for further researches that could be developed, comparing HRM practices among similar organizational fields or monitoring the longitudinal process of e-HRM to be integrated into daily HRM processes enacted by human players.

The article clearly presents some limitations, as it refers to a specific context and studies a particular historical phase of the firm in object; additionally, the MNEs represent a small minority of the business realities around the world. Another limitation is that, referring to a survey, a digital assessment and a pilot project might yield some confusion, but they are connected by the need to achieve different voices, languages, and dimensions embedded in HRM practices of a specific context seeking to promote digital innovation. To sum up, the authors have provided further evidence that it is possible to conceive “HRM as a socio-cultural artifact and understanding the field as a hologram, capturing the ambiguity of the concept and documenting the ‘multiple, shifting, competing, and more often than not, contingent identities”’ (Janssens and Steyaert, 2009, p. 152, quoting Keenoy, 1999, p. 5).

## Data Availability Statement

The raw data supporting the conclusions of this article will be made available by the authors, without undue reservation, to any qualified researcher.

## Ethics Statement

The studies involving human participants were reviewed and approved by the Compliance Program Supervisory Body ex Italian Decree Law 231/2001 and the Internal Control Committee of Comau S.p.A. Written informed consent to participate in this study was provided by the participants’ legal guardian/next of kin.

## Author Contributions

All authors contributed equally to constructing the conceptual framework, collecting the empirical data, analyzing the data, and writing the manuscript.

## Conflict of Interest

EF was employed by the company Comau S.p.A. The remaining authors declare that the research was conducted in the absence of any commercial or financial relationships that could be construed as a potential conflict of interest.

## Footnotes

1 https://www.businessroundtable.org/business-roundtable-redefines-the-purpose-of-a-corporation-to-promote-an-economy-that-serves-all-americans

2 About the ethics compliance, the studies involving human participants were reviewed and approved by Serena Sansonetti, member of the Compliance Program Supervisory Body ex Italian Decree Law 231/2001 and of the Internal Control Committee of Comau S.p.A.
