# Supplementary material for: HRM 4.0 and New Managerial Competences Profile: The COMAU Case
Source: Front Psychol. 2020 Nov 20;11:578251. doi: 10.3389/fpsyg.2020.578251 (PMC7714775; doi:10.3389/fpsyg.2020.578251)
Supplement: Supplementary file 1 [file Data_Sheet_1.pdf]

Digital Knowledge

Welcome to our Digital Knowledge survey

Dear colleague,

Comau is committed to innovation to meet challenges of a constantly developing market and the new requirements determined by the “digital revolution” .

In this new challenge, we need to adapt our skills and knowledge, leveraging at first what we have and then building up together for the future.

For this reason, you have been chosen to participate to this quick survey that will take very few minutes of your time.

This is not a professional evaluation of your skills but just a survey about how familiar you are with digital matters and your overall interest on this topic.
It is crucial for us to have your contribution.

Thanks for your time

* 1. Who are you?

Name and Surname [ ]

Country [ ]

* 2. How much are you interested on:

	No interest	I'm quite interested	I'm very interested
Application Development / Programming Languages	○	○	○
Internet of things	○	○	○
Cloud Technology	○	○	○
Data Management	○	○	○
Machine Learning / Artificial Intelligence	○	○	○
Digital User Experience	○	○	○
Digital Trends Outlook	○	○	○
Mobility and Connectivity	○	○	○

COMAU

Digital Knowledge

* 3. How would you evaluate your knowledge about:

	N/A	I have basic knowledge	I have some experience	I'm an expert
MES /PLM /MRP /ERP Systems	○	○	○	○
Programming Languages	○	○	○	○
Cloud	○	○	○	○
Communication API	○	○	○	○
Sensors knowledge	○	○	○	○
Edge Computing	○	○	○	○
Internet of Things Infrastucture	○	○	○	○
Data Management (Analysis, aggregation, security, database)	○	○	○	○
Machine Learning Algorithms	○	○	○	○
Artificial Intelligence Techniques	○	○	○	○
Digital twin concepts	○	○	○	○
ICT Infrastructure (Networking, wireless, Communication Protocols)	○	○	○	○
GUI Design	○	○	○	○

4. Is there any digital skill you master and that has not been mentioned?

COMAU

Digital Knowledge

* 5. Thinking about Digital transformation...

- ◯ that's not my favourite topic
- ◯ I would like to participate to a project
- ◯ I would like it to be part of my job
- ◯ I would like to be a digital champion

* 6. In your opinion which could be the future role of Comau in Digital transformation?

Follower — Leader

7. Any comment before leaving the survey?

Don't forget to keep your CV updated on SAP HR
